# Extremely Rare Neonatal Case With Pyloric Atresia, Heart Defects, Hypotonia, Jaundice, and Acidosis

**DOI:** 10.1002/ccr3.70727

**Published:** 2025-08-03

**Authors:** Saja Abouodeh, Yasmeen Alshami, Osama Hroub, Mohammad Hroub, Ahmad G. Hammouri, Ahmad Shaltaf, Wafa Shihadeh, Nimatee Dawod

**Affiliations:** ^1^ Faculty of Medicine Palestine Polytechnic University Bethlehem Palestine; ^2^ Faculty of Medicine Palestine Polytechnic University Hebron Palestine; ^3^ Radiology Department Al‐Ahli Hospital Hebron Palestine; ^4^ Ped Surgeon Ramallah Palestine; ^5^ Ped Surgery Fellow Ramallah Palestine; ^6^ General Surgery Resident Ramallah Palestine

**Keywords:** congenital ventricular septal defect, gastro‐duodenostomy in newborns, isolated pyloric atresia, metabolic acidosis in neonates, non‐syndromic neonatal intestinal obstruction, surgical management of pyloric atresia

## Abstract

Pyloric atresia (PA) is an exceptionally rare congenital cause of gastric outlet obstruction, often associated with syndromic conditions such as epidermolysis bullosa (EB). This case highlights a diagnostically challenging presentation of *non‐syndromic pyloric atresia* in a neonate, complicated by a moderate mid‐muscular ventricular septal defect (VSD), metabolic acidosis, and physiologic hydronephrosis, without any cutaneous manifestations. Initial hypotonia and respiratory irregularities diverted suspicion toward neurological or septic etiologies, delaying definitive diagnosis. Imaging, including upper gastrointestinal contrast study and echocardiography, confirmed the diagnosis of complete gastric outlet obstruction due to PA and coexisting VSD. The patient underwent successful gastro‐duodenostomy with resolution of symptoms and stable postoperative recovery. This case underscores the importance of maintaining a high index of suspicion for gastrointestinal obstruction in neonates with polyhydramnios and early feeding intolerance, even in the absence of classic syndromic features. Comprehensive, multidisciplinary evaluation—incorporating pediatric surgery, neonatology, and cardiology—is essential to address overlapping congenital anomalies and optimize outcomes. This report contributes to the limited literature on non‐syndromic PA with multisystem involvement.

## 1 Introduction

Pyloric atresia is an extremely rare congenital anomaly estimated to occur in at least 1 in 100,000 live births and accounts for less than 1% of all intestinal atresias [1, 2]. While it is often associated with epidermolysis bullosa (EB) or multiple intestinal atresia syndromes [2, 3], our patient represents a rare incident of non‐syndromic PA with very severe cardiac and metabolic complications that have seldom been documented [2, 4].

The case is particularly exciting due to diagnostic complexity, wherein the initial symptoms of hypotonia and respiratory distress diverted early clinical suspicion toward neurological or septic pathology. Eventually, the confirmation of complete gastric outlet obstruction through contrast studies was achieved together with echocardiographic findings of moderate mid‐muscular VSD, exposing the challenges in diagnosing and managing concurrent congenital anomalies [5, 6]. The other significant factor distinguishing this case from the more common syndromic presentations is the absence of cutaneous manifestations that are generally associated with EB [2, 3].

The clinical importance of this article lies in proving: (1) the diagnostic pitfalls in newborn PA with atypical features, (2) the complex relationship between gastrointestinal obstruction and systemic complications such as metabolic acidosis and hyperbilirubinemia [4, 7], and (3) the need for effective multidisciplinary cooperation among pediatric surgical, cardiology, and neonatology services to optimize results [4, 8]. It represents an addition to the meager literature regarding a non‐syndromic PA with extracutaneous manifestations while reinforcing the need for a thorough examination of any neonate presenting with polyhydramnios and feeding intolerance [1, 2].

## 2 Case Presentation

The patient is a 6‐day‐old infant; he was born at a gestational age of 36 weeks and 5 days through normal vaginal delivery with a birth weight of 2740 g. Except for polyhydramnios, the pregnancy was uneventful. The patient was hypoactive at birth, had a weak cry, and was hypotonic with irregular respiration. Immediate stimulation and bag‐mask ventilation were done, which improved respiration and heart rate reached over 100 beats per minute; the patient's skin became pink, but his tone remained poor initially. Apgar scores were 6 and 8. A cord blood gas sample was taken to show a pH of 7.04, Pco2 27 mmHg, HCO_3_–9.9 mmol/L, and base excess −18.7, and the baby was discharged against medical advice.

He was brought to the Emergency department with a history of vomiting after feeding, which was not associated with fever or other complaints. Physical examination showed a soft, lax abdomen. The patient was well appearing and did not show signs of distress.

The infant was transferred to the Neonatal Intensive Care Unit as a case of suspected sepsis or possible underlying neurological disease. Blood culture was taken, and empiric intravenous antibiotics (ampicillin and gentamicin) were initiated. The baby later developed recurrent vomiting. Ultrasound showed mild right‐sided hydronephrosis. Antibiotics were escalated to intravenous meropenem and amikacin. Blood cultures remained negative.

Abdominal ultrasound conclusion:

- There is no evidence of hypertrophic pyloric stenosis.
- Mild right‐sided hydronephrosis (renal pelvis 0.4 cm), likely physiological.
- Normal relationship of the superior mesenteric artery to the superior mesenteric vein.
- No signs of intestinal malrotation or bowel obstruction.

**FIGURE 1:**
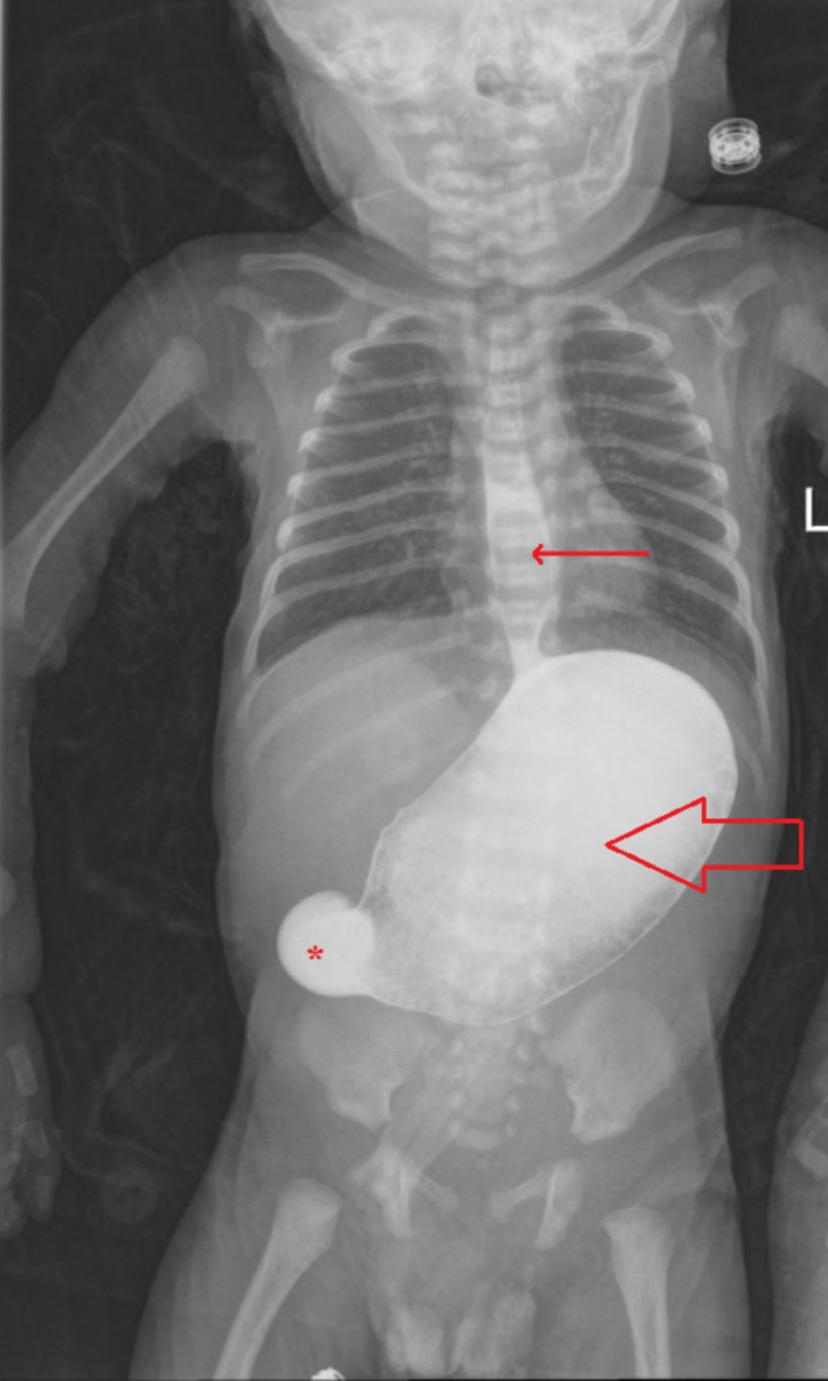
Upper gastrointestinal study demonstrating the distended contrast‐filled stomach (open arrow) with no contrast seen passing beyond the pylorus (asterisk) even after 2 h. The contractions are seen refluxing into the distal esophagus (solid arrow) during the examination.

An upper gastrointestinal study was done at a private radiology center, which confirmed the diagnosis of gastric outlet obstruction. The swallowing mechanism and the esophagus were normal, but the stomach was distended with no contrast passing distally even after 2 h (as demonstrated in Figure 1). During the study, the patient had episodes of reflux and vomiting, with contrast refluxing into the distal esophagus.

**FIGURE 2:**
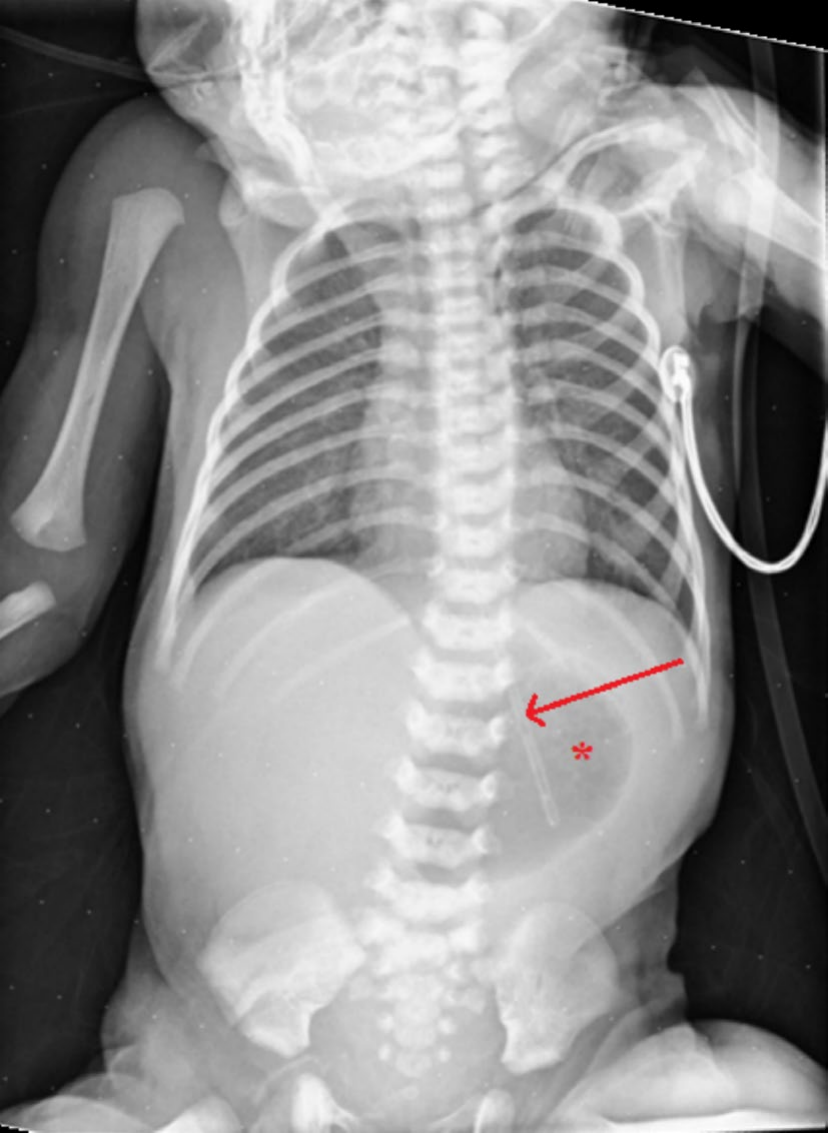
Pre‐operative Chest and abdomen AP x‐ray showing a gas‐filled dilated stomach (Red Asterisk) with a paucity of bowel gas in the small and large bowel. The distal end of a nasogastric tube is marked with the red arrow.

A pre‐operative chest and abdominal x‐ray had shown a gas‐filled dilated stomach with a paucity of distal bowel gas and the nasogastric tube tip in place (Figure 2).

**FIGURE 3:**
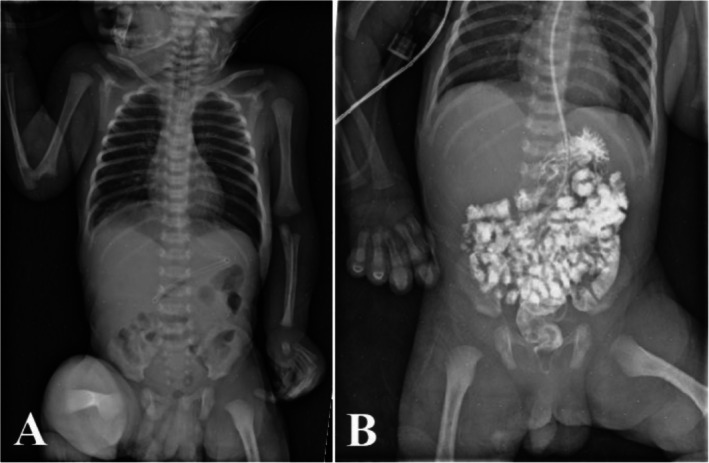
Post‐operative images obtained for the patient showing an unremarkable bowel gas distribution pattern (A). The contract is seen filling the non‐dilated bowel lobes (B), indicating resolution of the pre‐operative pyloric obstruction.

Echocardiography revealed:

- Normal cardiac chambers and function.
- Moderate mid‐muscular ventricular septal defect with left‐to‐right shunt, pressure gradient of 30 mmHg.
- Small patent foramen ovale.
- Small patent ductus arteriosus.
- No coarctation of the aorta.
- Trace tricuspid regurgitation and physiologic pulmonary artery stenosis.

## 3 Differential Diagnosis, Investigations and Treatment

Diagnosis of pyloric atresia was established. The infant underwent exploratory laparotomy, direct visualization confirmed the diagnosis, and Gastro‐Duodenostomy was done by making an anastomosis between the prepyloric region and the duodenum. Postoperatively, the baby was admitted to the Pediatric ICU for monitoring.

The infant was kept NPO with nasogastric tube drainage. Total parenteral nutrition was started and titrated. Gradual enteral feeding was initiated, starting with 30 mL every 3 h, increasing by 3 mL each interval until full feeds were achieved (55–60 mL every 3 h). NGT was later removed, and oral feeding was well tolerated.

A follow‐up abdominal x‐ray showed unremarkable bowel gas distribution and contrast filling the non‐dilated bowel loops, confirming resolution of the obstruction (Figure 3).

The infant received intravenous cefotaxime and metronidazole. Phototherapy was given initially for indirect hyperbilirubinemia (total serum bilirubin 13.6 mg/dL). Renal ultrasound was done again, showing mild hydronephrosis on the left side (6.5 mm), likely physiologic.

The patient remained hemodynamically stable throughout admission and was followed up by the pediatric surgery and neonatology teams. Plans were made for eventual transfer to the Pediatric Ward after stabilization.

## 4 Conclusion and Results (Outcome and Follow‐Up)

This case has rare and diagnostically challenging neonatal presentations of pyloric atresia (PA), with unique complexities in both clinical presentations and management, contrasting significantly with the standard cases. The infant was, therefore, a composite of findings: a moderate mid‐muscular ventricular septal defect (VSD), the physiologic hydronephrosis, hypotonia, and metabolic acidosis constellation of findings, which do not manifest the cutaneous symptoms associated with Epidermolysis Bullosa (EB) syndrome syndromic associations. The case is further unique from the usual presentation since there were no findings of EB or other syndromic features, which often accompany PA.

Diagnostic attempts became even more challenging when this infant's initial presentation of hypotonia and some respiratory irregularities diverted early suspicion toward neurological or septic causes. This definitive diagnosis was established based on upper GI contrast study and echocardiography, highlighting the significance of a systematic, multidisciplinary approach in neonates with feeding intolerance and congenital anomalies.

Management was uniquely arduous because VSD necessitated cardiac monitoring, the patient required TPN nutritional support while metabolic derangements and hyperbilirubinemia were being treated, and surgical correction (gastro‐duodenostomy) simultaneously addressed the other medical problems. Symptomatic improvement without any long‐term sequelae supports the effectiveness of urgent surgical treatment performed cooperatively by pediatric surgery, cardiology, and neonatology teams. Consequently, this case stands out due to its atypical presentation, the absence of classic syndromic associations, and the need for a fine balance in managing various congenital anomalies at the same time. It is an addition to the scant literature on non‐syndromic PA with involvement of the heart, providing a reminder to be extra vigilant in watching neonates with polyhydramnios and vomiting and the necessity of postnatal evaluation that may uncover rare, coexisting conditions.

This infant, who underwent successful gastro‐duodenostomy, had complete resolution of gastric outlet obstruction, was noted with no complications in postoperative recovery, progressing enteral feeding, and a short course of phototherapy for hyperbilirubinemia (peak bilirubin 13.6 mg/dL). Other anomalies, such as a moderate mid‐muscular VSD (30 mmHg gradient) and mild bilateral hydronephrosis, were still hemodynamically insignificant, requiring surveillance only. With the absence of Epidermolysis Bullosa or syndromic features, this presented as an unusual variant of non‐syndromic PA with multisystem involvement. At discharge, the infant displayed excellent outcomes of surgical success, normalized metabolic parameters, and stable cardiac and renal functions that testified to the appropriate multidisciplinary neonatal care provision of the complex case.

## 5 Discussion

A 6‐day‐old neonate presented with gastric outlet obstruction because of pyloric atresia (PA), which occurs rarely in 1 out of 100,000 live births [1]. Pyloric atresia causes polyhydramnios because fetal swallowing becomes impeded when there is gastrointestinal blockage [3]. Feeding intolerance alongside vomiting and failed contrast upper gastrointestinal study (UGI) test results supported the diagnosis of complete gastric outlet obstruction, which verified the diagnosis of pyloric atresia.

PA clinical findings appeared in the neonate as non‐bilious vomiting with abdominal distension and diminished growth [9]. A complete obstruction shown on UGI study, combined with normal ultrasound findings excluding hypertrophic pyloric stenosis, helped differentiate PA from antral webs and malrotation as possible gastric outlet obstruction causes [5]. Systematic assessment of these cases becomes more important since congenital defects such as moderate ventricular septal defect and mild hydronephrosis were detected. PA exists as an isolated condition, but it sometimes occurs as a component of Epidermolysis bullosa (EB) or multiple intestinal atresia syndromes [9]. The evaluation did not demonstrate either skin manifestations or syndromic characteristics.

Surgery is the preferred therapy for PA because gastro‐duodenostomy restores gastrointestinal continuity [9]. Infants gradually tolerate enteral feeding after the gastro‐duodenostomy is performed. During the first postoperative period, the use of total parenteral nutrition (TPN) is critical because it supports nutritional needs and allows anastomotic healing [4]. The antibiotic combination of cefotaxime and metronidazole protected the infant from postoperative infections while the infant maintained a prolonged NPO status [8].

The combination of mid‐muscular VSD and physiologic hydronephrosis during this case shows why it is essential to conduct detailed cardiac and renal evaluations for neonates born with congenital gastrointestinal anomalies [3]. Long‐term patient monitoring is essential to assess both spontaneous VSD closure and potential complications since the VSD demonstrates no major hemodynamic impacts [6]. The temporary hyperbilirubinemia developed because of dehydration and was treated successfully with phototherapy as a standard therapy for infants with feeding problem [7].

## Author Contributions

**Saja Abouodeh:** conceptualization, data curation, formal analysis, methodology, software, supervision, validation, writing – original draft. **Yasmeen Alshami:** conceptualization, data curation, investigation, project administration, resources, software, validation, writing – original draft. **Osama Hroub:** formal analysis, investigation, methodology, project administration, resources, supervision, visualization, writing – review and editing. **Mohammad Hroub:** conceptualization, data curation, funding acquisition, methodology, resources, software, supervision, writing – original draft. **Ahmad G. Hammouri:** project administration, supervision, validation, visualization, writing – review and editing. **Ahmad Shaltaf:** methodology, resources, supervision. **Wafa Shihadeh:** investigation, software, validation. **Nimatee Dawod:** funding acquisition, resources, supervision.

## Consent

Written informed consent was obtained from the patient for the publication of this case report.

## Conflicts of Interest

The authors declare no conflicts of interest.

## Data Availability

The data used to support the findings of this study are included in the article.
